# Fast Removal of Naphthol Blue Black B Dye from Water Using Polyethyleneimine Functionalized Zinc, Iron, and Manganese Porphyrinic Complexes: Structural Characterization, Kinetic, and Isotherms Studies

**DOI:** 10.3390/polym17111494

**Published:** 2025-05-28

**Authors:** Sahar Y. Rajeh, Aljazi Abdullah Alrashidi, Raoudha Soury, Mahjoub Jabli

**Affiliations:** 1 Chemistry Department, College of Science, University of Ha’il, Ha’il 81451, Saudi Arabia; s.rajeh@uoh.edu.sa (S.Y.R.); a.alrashedy@uoh.edu.sa (A.A.A.); 2 Department of Chemistry, College of Science, Majmaah University, Al-Majmaah 11952, Saudi Arabia

**Keywords:** Zn(TMP), Fe(TPP)Cl, Mn(TPP)Cl, polyethyleneimine, adsorption, naphthol blue black B

## Abstract

In the present work, meso-tetrakis(2,4,6-trimethylphenyl) porphyrinato)zinc(II): ([Zn(TMP)] **(1)**, meso-tetrakis-(tetraphenyl)porphyrin iron(III))chloride): [Fe(TPP)Cl] **(2)**, and meso-tetrakis(phenyl)porphyrin manganese(III) chloride): [Mn(TPP)Cl] (3) were synthesized. Then, the three prepared porphyrinic complexes **(1**–**3)** were functionalized with branched polyethyleneimine (PEI). The prepared complexes were thoroughly analyzed using several analytical techniques, including ^1^H NMR, FT-IR, UV-vis, XRD, XRF, TGA-DTA, SEM, and EDX. The presence of sharp main peaks at 2θ between 10° and 80°, in XRD analysis, for all studied compounds suggested the crystalline nature of the porphyrinic complexes. The morphological properties of the porphyrininc complexes were significantly affected by the chemical modification with polyethyleneimine. EDX result confirmed the complexation of zinc, iron, and manganese metals with the porphyrinic core. The increase in carbon and nitrogen contents after the addition of polyethyleneimine to the complexes **(1**–**3)** was noticeable. After thermal decomposition, the total mass loss was equal to 92.97%, 66.77%, and 26.78% for complexes **(1)**, **(2)**, and **(3)**, respectively. However, for the complex **(1)**-PEI, complex **(2)**-PEI, and complex **(3)**-PEI, the total mass losses were 83.12%, 81.88%, and 35.78%, respectively. The synthetic compounds were additionally utilized for the adsorption of Naphthol blue black B from water. At optimum adsorption conditions (T = 20 °C, time = 60 min, pH = 5), the highest adsorption capacities were 154 mg/g, 139 mg/g, and 119 mg/g for complex **(3)**-PEI, complex **(2)**-PEI, and complex **(1)**-PEI, respectively. The adsorption mechanism followed the pseudo second order, the Freundlich, and the Temkin models. The results indicated that the adsorption process is reliant on chemical interactions. It was also governed by intraparticular diffusion and other kinetic phenomena.

## 1. Introduction

Porphyrinic complexes are an important class due to their versatile properties and features [1,2]. Structurally, porphyrin is composed of four pyrrolic units linked in a coplanar fashion by four methane bridges that give a planar macrocyclic structure. It has an extended conjugated 18 π-electron system, which is responsible for its aromatic behavior. Indeed, the free-base porphyrin reacts with metal cations to form complexes with a variety of conformations, depending especially on the size of metal cations, the axial ligands, the size of peripheral substituents, etc. [3,4]. The properties of metalloporphyrins can be controlled by the fine-tuning of the out-of-plane distance in the porphyrin cavity. Owing to these intriguing features, porphyrins and metalloporphyrins have been widely used in several interesting fields, including theragnostic [5,6,7,8,9,10,11,12], opto-electronics [13,14,15], photocatalysis [16], and other important applications [17,18]. In particular, there is a considerable increase in the usage of metalloporphyrin-based materials for the removal of several harmful contaminants from water in order to limit toxic substance use and manufacture. In previous work, we and other researchers showed that the characteristics, and hence the application, of a metalloporphyrin complex may be altered by modifying the metal [19,20,21,22], the axial ligand(s) coupled to the metal [23,24], and the porphyrin perimeter [25,26,27]. Recently, some investigations have concentrated on the construction of new porphyrins modified with polymers formed predominantly through intermolecular coordination and hydrogen-bonding interactions [28,29].

Polyethyleneimine, which is an organic polyamine polymer, is considered one of the most important examples of cationic polymers. It is rich in amine groups. These amine groups could rapidly interact with several organic and inorganic pollutants. In the literature, some works have reported on the chemical modification of a series of compounds using polyethyleneimine. For instance, Teng et al. [30] used polyethyleneimine to modify polyvinylidene fluoride membrane. The modified membranes exhibited good ability to adsorb organic dyes and heavy metal ions from water and reject them via filtration. Lang et al. [31] fabricated a polyethyleneimine/nanocellulose/MXene/loofah composite aerogel. The aerogel was able to efficiently adsorb heavy crude oil and can selectively adsorb methylene blue, malachite green, Congo red, and methyl orange from mixed dye systems. Yan et al. [32] synthesized a composite aerogel via the lyophilization of cellulose modified with polyethyleneimine and cadmium sulfide. This composite demonstrated exceptional efficacy in degrading methyl orange and methylene blue. In our previous work, we reported the extraction and chemical functionalization of cellulose from *Echinops bannaticus* leaves. The extracted cellulose was functionalized with Poly (diallyldimethylammonium chloride) and branched polyethyleneimine. The modified samples demonstrated good adsorption capacities of acid blue 25, an anionic dye [33].

Herein, we report, first, the synthesis and the characterization of the following complexes **(1**–**3)**; meso-tetrakis(2,4,6-trimethylphenyl) porphyrinato)zinc(II): ([Zn(TMP)] **(1)**, meso-tetrakis-(tetraphenyl)porphyrin iron(III))chloride): [Fe(TPP)Cl] **(2)**, meso-tetrakis(phenyl)porphyrin manganese(III) chloride): [Mn(TPP)Cl] **(3)**. Then, the prepared porpyrinic complexes **(1**–**3)** were functionalized with branched polyethyleneimine in order to impart reactive amino groups and improve their adsorption capacities toward anionic species. All prepared materials were thoroughly analyzed using several analytical techniques, including 1H NMR, FT-IR, UV-vis, EDX, XRF, SEM, TGA-DTA, and XRD. Materials were further used for the adsorption of Naphthol blue black B from water. Factors influencing the adsorption mechanism, such as pH, time, initial Naphthol blue black B concentration, and temperature, were assessed. The adsorption results were fitted to theoretical kinetic and isotherm models in order to understand the adsorption phenomenon. The thermodynamic parameters were also calculated.

## 2. Experimental

### 2.1. Reagents, Materials, and Apparatus

Branched polyethyleneimine solution (50 wt.% in H_2_O, average Mw ~750,000) was purchased from Sigma Aldrich, S. Louis and Burlington, MA, USA and used for the chemical modification of the porphyrinic compounds. Naphthol blue black B (M.W = 616.5 g/mol, purity = 85%), an anionic dye, was used as the adsorbate during adsorption experiments. The pH values of the solutions were adjusted using sodium hydroxide and sulfuric acid solutions. All other used solvents or reagents were laboratory grade. An Ultrashield spectrometer, Quassim, Saudi Arabia (CDCl_3_ is used as solvent, and trimethylsilane is the reference) was used to record (^1^H)-NMR spectra. A Perkin Elmer Spectrum FT-IR (Monastir, Tunis) was used to provide FT-IR spectra. The absorption measurements were collected using a diluted sample solution in dichloromethane (Sigma Aldrich, USA, ST) The morphological characteristics of the porphyrinic compounds under study were described using a JEOL JSM-5400 SEM (Quassim, Saudi Arabia). An X-ray diffraction (XRD) pattern was obtained using PANalytical X’Pert PRO MPD equipment (Quassim, Saudi Arabia). The samples were evaluated within a 2ϴ range (10 to 90 degrees). Thermogravimetric data (TGA/DTA) were collected in an air flow with a heating rate of 10 °C per minute. Thermal events were investigated using a platinum crucible and the NE-TZSCH STA 449F3 instrument (Quassim, Saudi Arabia).

### 2.2. Synthesis of Complexes **1**–**3**

The meso-tetrakis(2,4,6-trimethylphenyl) porphyrinato)zinc(II): complex **(1)**, meso-tetrakis-(tetraphenyl) porphyrin iron(III)chloride: complex **(2)** and meso-tetrakis-(tetraphenyl) porphyrin manganese(III)chloride: complex **(3)** were synthesized following a method reported in our previous studies [34,35,36].

### 2.3. Synthesis of Complex (1)-PEI: Meso-Tetrakis(2,4,6-trimethylphenyl) Porphyrinato)zinc(II) Functionalized with Branched Polyethyleneimine: [Zn(TMP)]-PEI

[Zn(TMP)] (500 mg, 0.591 mmol) and excess branched PEI polymer solution were mixed in dichloromethane (10 mL) overnight at 25 °C. The hue of the solution shifted from pink to purple. The complex [Zn(TMP)]-branched polyethyleneimine was obtained as a pink powder. UV/Vis [CH_2_Cl_2_, λ_max_ in nm (log Ɛ)]: 424 (4.85), 554(3.68), 603(3.25).

### 2.4. Synthesis of Complex (2)-PEI: Meso-Tetrakis-(tetraphenyl) Porphyrin Iron(III)chloride Functionalized with Branched Polyethylenemine: [Fe(TPP)Cl]-PEI

[Fe(TPP)Cl] (500 mg, 0.747 mmol) and an excess of branched PEI polymer solution were mixed in dichloromethane (10 mL) at 25 °C overnight. The color of the solution turned purple. The complex [Fe(TPP)Cl]- branched PEI was obtained as a purple powder. UV/Vis [CH_2_Cl_2_, λ_max_ in nm (log Ɛ)]: 425 (4.98), 515(4.02).

### 2.5. Synthesis of the Complex (3)-PEI: Meso-Tetrakis-(tetraphenyl) Porphyrin Manganese(III)chloride Functionalized with Branched Polyethyleneimine: [Mn(TPP)Cl]-PEI

[Mn(TPP)Cl] (500 mg, 0.749 mmol) and an excess of branched PEI polymer solution were mixed for 12 h at 25 °C in dichloromethane (10 mL). The solution changed color from green to purple. The complex [Mn(TPP)Cl]-PEI was produced as a purple powder. UV/Vis [CH_2_Cl_2_, λ_max_ in nm (log Ɛ)]: 375 (3.97), 411 (4.74), 580(4.97), and 619(5.63).

### 2.6. Adsorption Experiments

An Erlenmeyer flask containing a mixture of 0.001 g of non-modified or functionalized porphyrinic complexes and 10 mL of Naphthol blue black B solution was continuously stirred (125 rpm) for a period of 2 h. Then, the contents of the flasks were filtered using Whatman filter paper, Riyadh, Saudi Arabia. The absorbance of the resulting solutions was measured using a UV-Vis spectrophotometer (Riyadh, Saudi Arabia) at the maximum wavelength absorption (λ_max_ = 610 nm). The adsorption capacities were further calculated. The studies were designed to test the effects of various contact periods (ranging from 0 to 120 min), Naphthol blue black B concentrations (ranging from 0 mg/L to 600 mg/L), and temperatures (22 °C to 53 °C).

## 3. Results

### 3.1. Sample Characterizations

#### 3.1.1. FT-IR Spectroscopy

**Figure 1:**
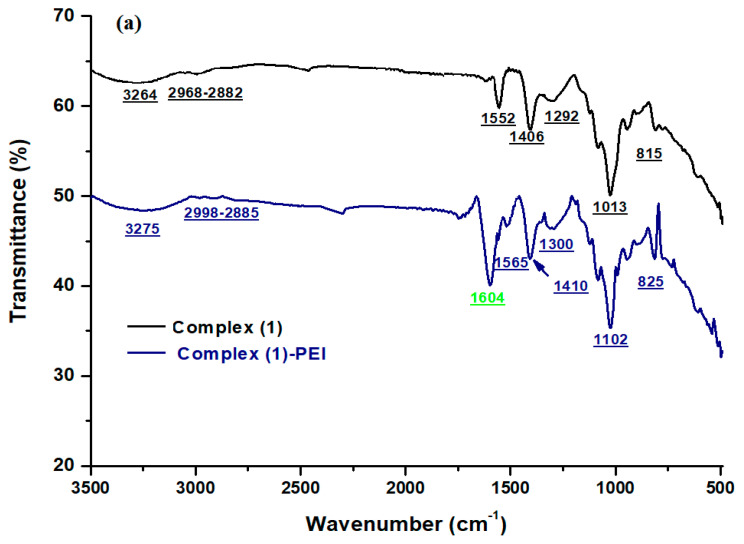

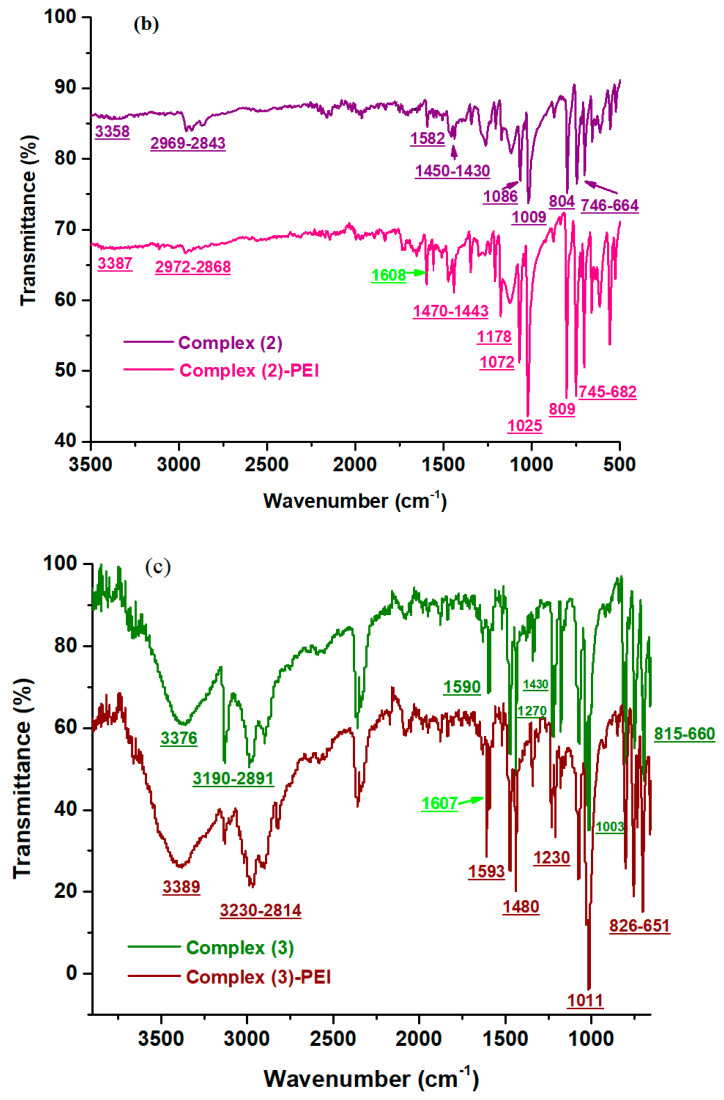
FT-IR spectra of (**a**) complex **(1)** and complex **(1)**-PEI, (**b**) complex **(2)** and complex **(2)**-PEI, (**c**) complex **(3)** and complex **(3)**-PEI.

The FT–IR spectra of complexes **(1**–**3)** and the modified complexes are given in Figure 1. For complexes **(1**–**3)**, the spectra show the presence of the main peaks of metalloporphyrins. The C-H group of the porphyrinic core is detected around 3264–3380 cm^−1^. The ν (C-N) peak is detected between 1430 and 1500 cm^−1^. The ν(C-C) peak is seen at 1590–1552 cm^−1^. The absorption peak for the vibration v (C-C) is around 1102–1230 cm^−1^. The vibration mode δ (CCH) of the porphyrin core is located at around 1008 cm^−1^. The absorption peak at 810 cm^−1^ corresponds to the v (C-C) phenyl group [37,38]. After chemical functionalization of the porphyrinic complexes **(1**–**3)** with branched polyethyleneimine, new absorption peaks appear at 1604, 1608, and 1607 cm^−1^ for complex **(1)**-PEI, complex **(2)**-PEI, and complex **(3)**-PEI, respectively. These absorption peaks can be assigned to the amine groups of polyethyleneimine [39]. This result confirms that the prepared porphyrinic complexes chemically interact with the polymer through amine groups.

#### 3.1.2. Optical Spectroscopy

**Figure 2:**
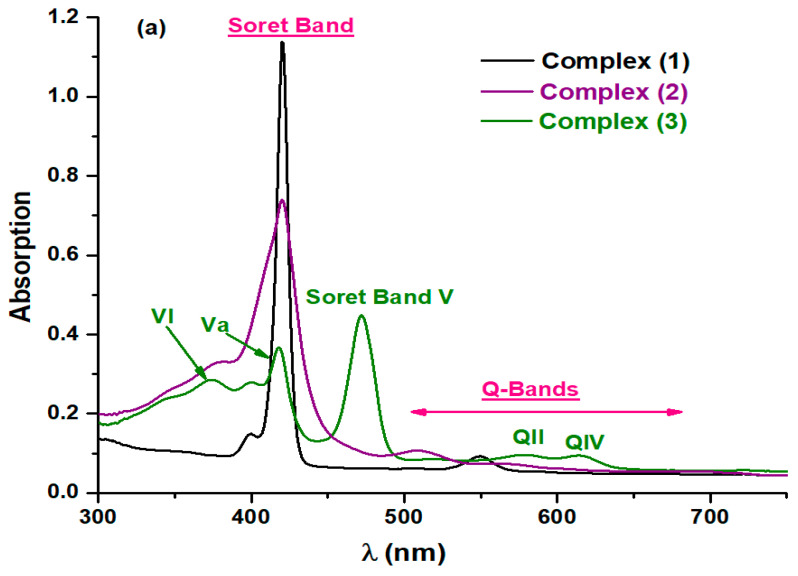

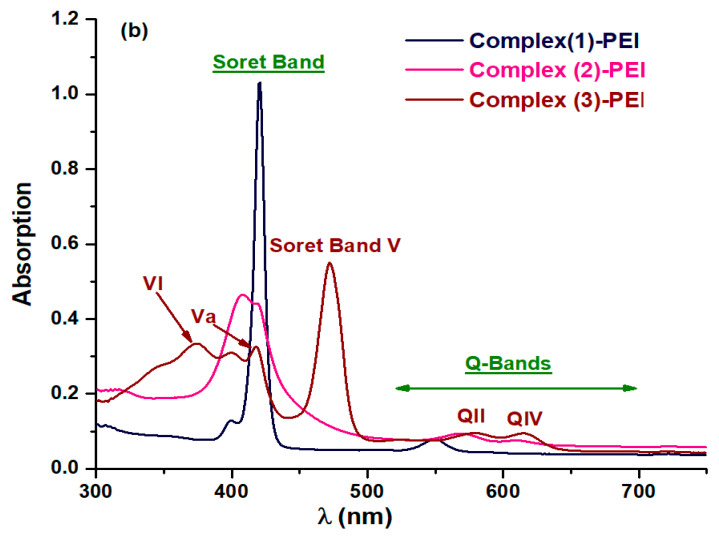
Spectroscopic data of: (**a**); complexes **(1**–**3)** and (**b**); complex **(1)**-PEI, complex **(2)**-PEI, and complex **(3)**-PEI in dichloromethane.

The UV-visible spectra of the complexes **(1**–**3)** and the modified complexes were studied in dichloromethane at a concentration of ca 10^−6^ M. Figure 2 elucidates the spectroscopic data of complex **(1**–**3)** and the modified ones. As observed, the change in the spectral profile is due to the presence of Zn, Fe, and Mn metals. We noticed that the spectrum of complex **(1)** exhibits a very intense band known as the Soret band, allowing the transition of the electrons from the ground state S0 to the second excited state S2 at 422 nm. In addition, two less intense absorption bands are observed at 550 nm and 596 nm, which are known as Q-Bands corresponding to the transition of electrons from the ground state to the first excited state S1 [30,31,34]. Regarding complex **(2)**, the UV-vis spectrum reveals the presence of a Soret band at 421 nm and one Q-band at 510 nm [35]. We observe significant changes for complex **(3)** compared to complex **(1)** and **(2)**. This is related to the manganese metal [36]. Complex **(3)** contains a hypertype electronic spectrum that includes a half-vacant metal orbital with symmetry, such as [dπ:dxz and dyz] [36]. The Soret band V, or the strongest band, is identified at 471 nm. This band results from the transfer of the porphyrin’s a1u(π) and a2u(π) orbitals to manganese orbitals, such as [dπ: dxz and dyz]. The two less intense bands than the Soret band, the VI and Va bands, are detected at 372 nm and 408 nm, respectively. In addition, there are two Q-bands, the QII and QIV bands, observed at 577 nm and 615 nm, respectively. After chemical modification of the complexes **(1**–**3)** with polyethyleneimine, we observe a slight bathochromic shift in the values of λ_max_ of the bands. This suggests the interaction of complexes **(1**–**3)** with the polyethyleneimine polymer.

#### 3.1.3. XRD Analysis

**Figure 3:**
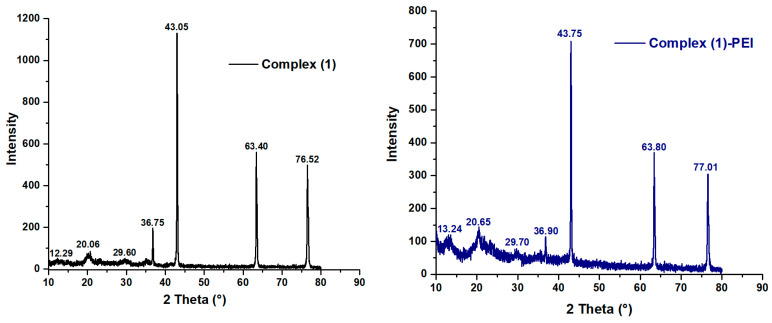

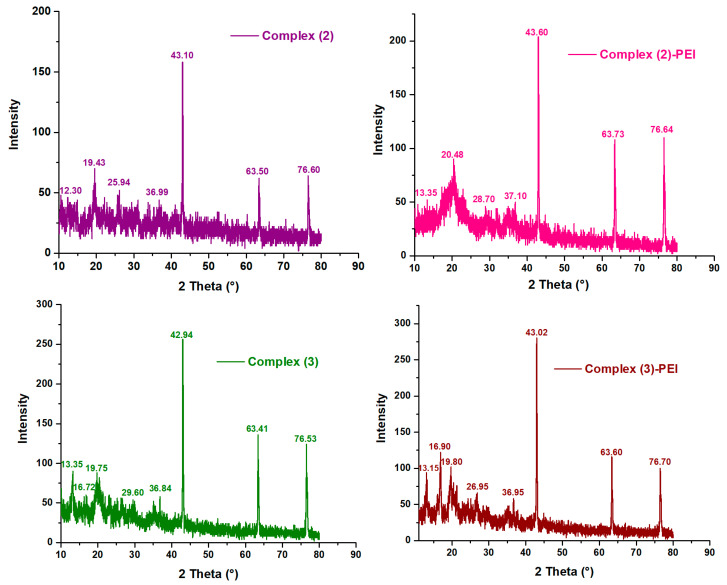
XRD patterns of complexes **(1–3),** complex **(1)**-PEI, complex **(2)**-PEI, and complex **(3)**-PEI.

Figure 3 shows the XRD patterns of complex **(1**–**3)** and modified complexes. As shown, the complex **1** reveals sharp peaks observed at 2θ = 12.29°, 20.05°, 26.60°, 36.75°, 43.05°, 63.40°, and 76.52°. However, the complex **2** exhibits a series of peaks observed at 2θ = 12.30°, 19.43°, 25.94°, 36.99°, 43.10°, 63.50°, and 76.60°. The complex **3** displays a series of peaks observed at 2θ = 13.35°, 16.72°, 19.75°, 29.60°, 36.84°, 42.94°, 63.41°, and 76.53°. These peaks point to the crystalline character of the porphyrinic complexes [40,41]. In addition, we find a slight displacement of the position of the principal peaks for the modified complexes **(1**–**3)** compared to the unmodified ones. This shifting suggests again the chemical interaction between the complexes **(1**–**3)** and polyethyleneimine.

#### 3.1.4. SEM and EDX Analysis

**Figure 4:**
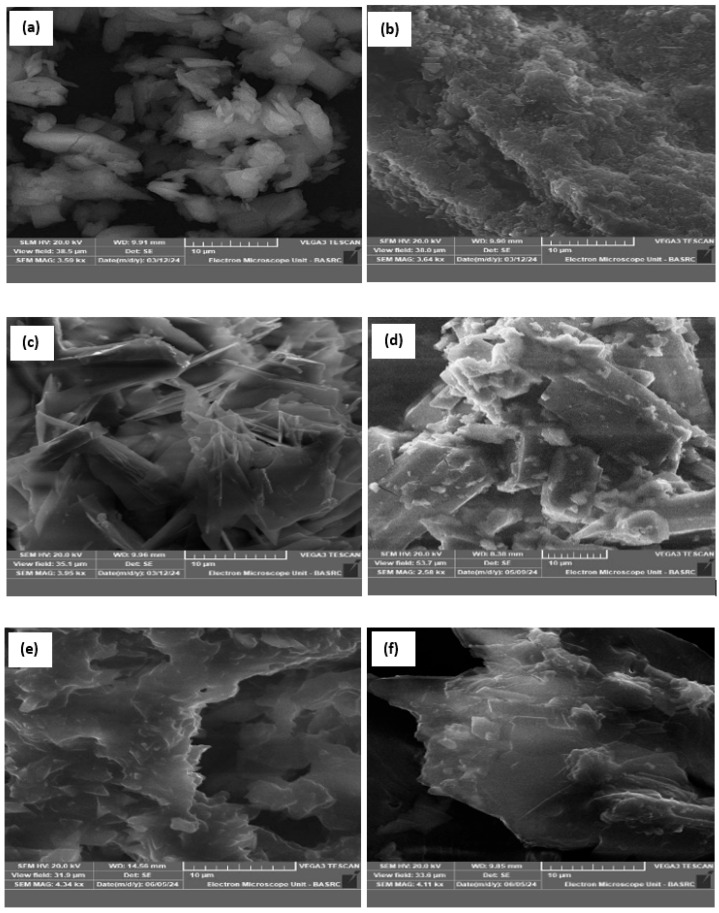
SEM images of: (**a**) complex **(1)**, (**b**) complex **(2),** (**c**) complex **(3),** (**d**) complex **(1)**-PEI, (**e**) complex **(2)**-PEI, and (**f**) complex **(3)**-PEI.

Figure 4 gives the morphological characteristics of the complexes **(1**–**3)** and modified complexes. As observed, the chemical modification of the prepared complexes clearly affects the morphological properties of the porphyrinic complexes. In particular, the particles become more agglomerated due to the chemical interaction between polyethyleneimine and the porphyrinic complexes.

**Figure 5:**
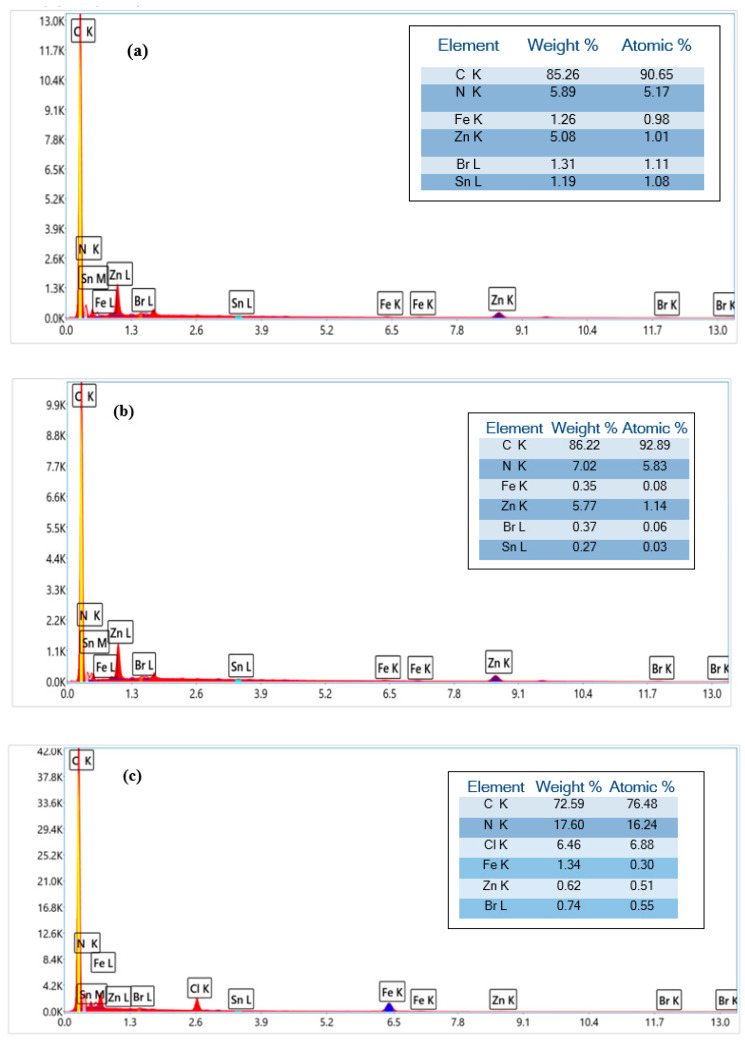

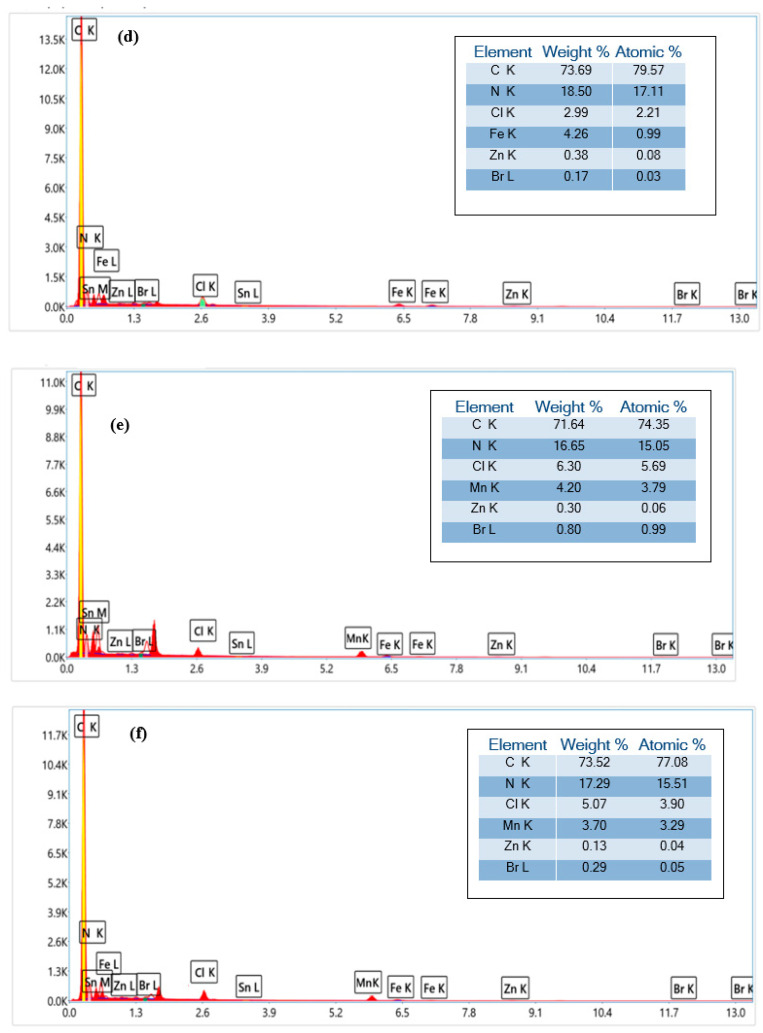
EDX result of: (**a**) complex **(1)**, (**c**) complex **(2)**, (**e**) complex **(3)**, (**b**) complex **(1)**-PEI, (**d**) complex **(2)**-PEI, and (**f**) complex **(3)**-PEI.

The EDX data support the complexation of zinc, iron, and manganese metals with the porphyrinic core (Figure 5). As expected, an increase in carbon and nitrogen contents after the addition of PEI polymer to the complexes **(1**–**3)** is visible. This confirms the interaction between the porphyrinic complexes and polyethyleneimine.

#### 3.1.5. X-Ray Fluorescence Analysis

The X-ray fluorescence (XRF) data of complexes **(1**–**3)** and modified complexes, showing the relative abundance of elements, are summarized in Table 1. The results show that the metals Zn, Fe, and Mn are present in the complexes **(1**–**3)** with relative amounts of 20.63%, 52.60%, and 70.59%, respectively. Other elements, such as Cl, Na, Al, Ca, K, Ti, Nb, Mo, In, Hf, Sx, and V are also found in the prepared complexes. As observed, the relative abundance of these elements is slightly changed after chemical modification with polyethyleneimine. This result can be explained by the electron transfer pathway between the metals and the nitrogen atom in polyethyleneimine polymer [42,43].

#### 3.1.6. Thermal Analysis

**Figure 6:**
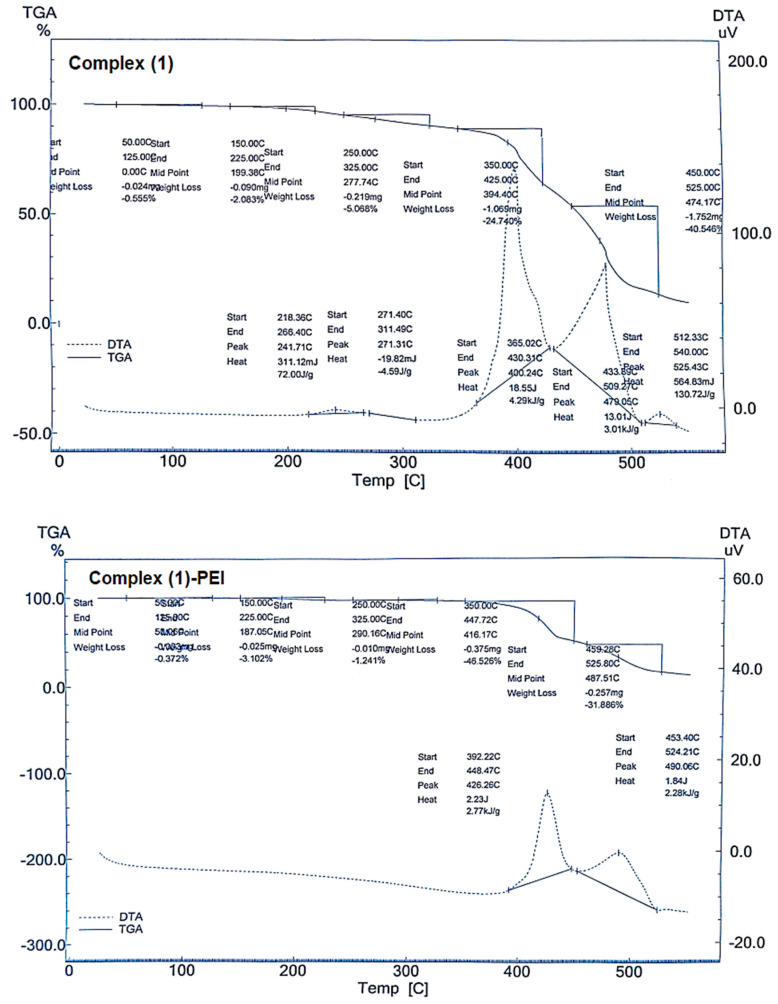

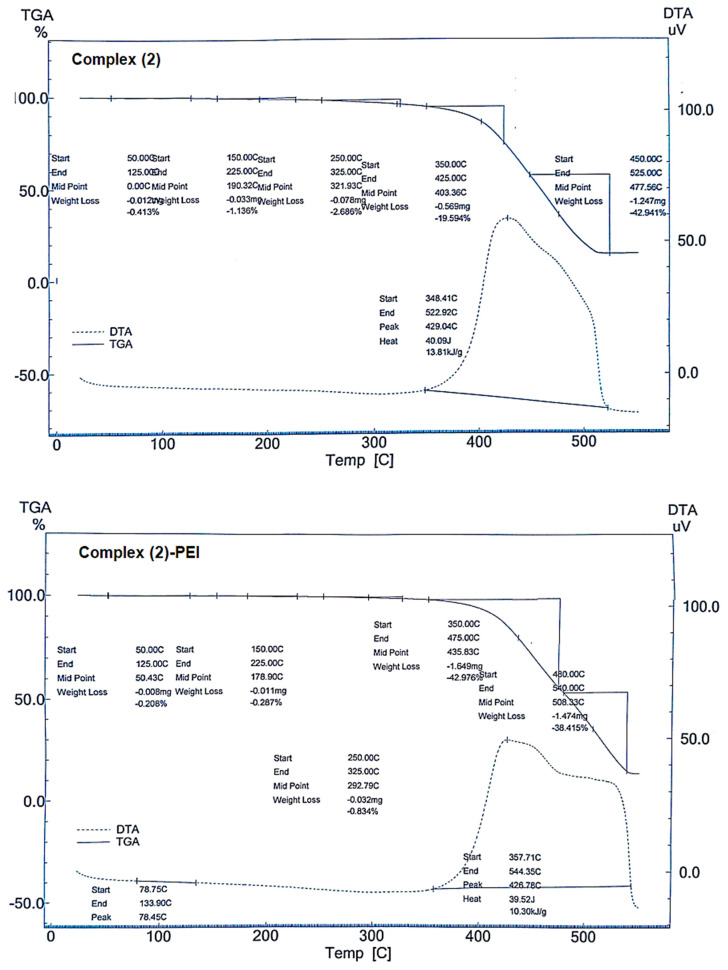

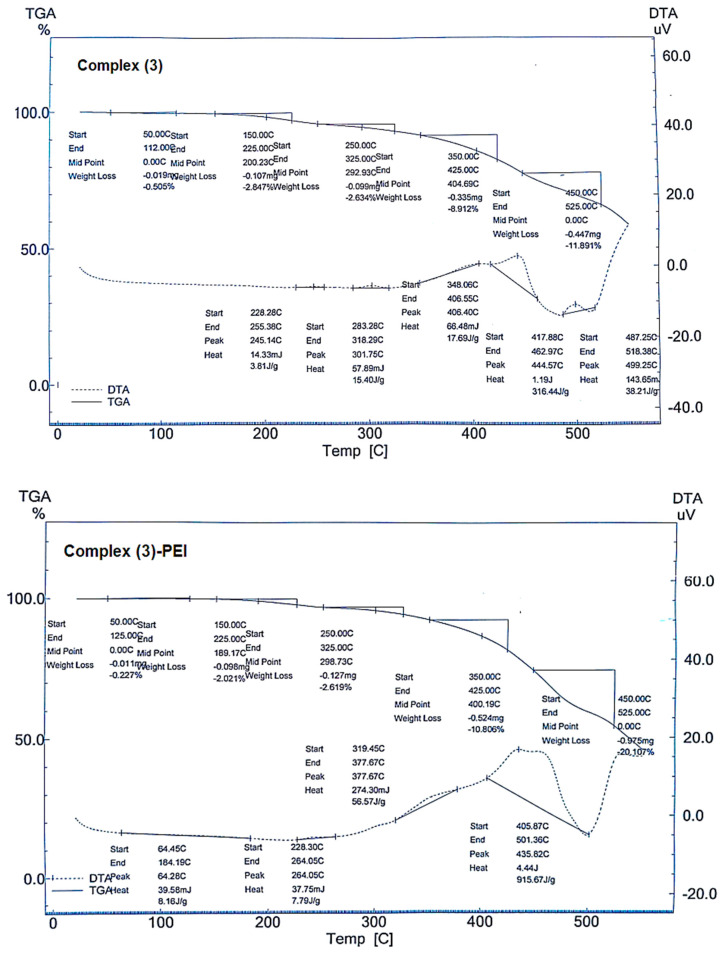
TGA/DTA curves of complexes **(1**–**3)** and modified complexes.

The thermal analysis of complexes **(1**–**3)** and modified complexes is shown in Figure 6. All the obtained complexes show thermal processes at temperatures below 100 °C, indicating the release of adsorbed water [44]. The total mass reduction is 72.97%, 66.77%, and 26.78% for complexes **1**, **2**, and **3**, respectively. For the modified complexes, five occurrences are noticed in the range [50–450 °C], with total mass loss of 83.12% (complex **1**-PEI), 81.88% (complex **2**-PEI), and 35.78% (complex **3**-PEI). The increase in the mass loss for the modified complexes confirms the chemical interaction of the porphyrinic complexes modified with polyethyleneimine. This trend also justifies the existence and degradation of the polymeric material. The thermal events indicate that the non-modified compounds are more thermostable than the functionalized ones.

### 3.2. Application of the Prepared Complexes in Naphthol Blue Black B Adsorption

#### 3.2.1. Effect of the Experimental Parameters on the Adsorption Mechanism

**Figure 7:**
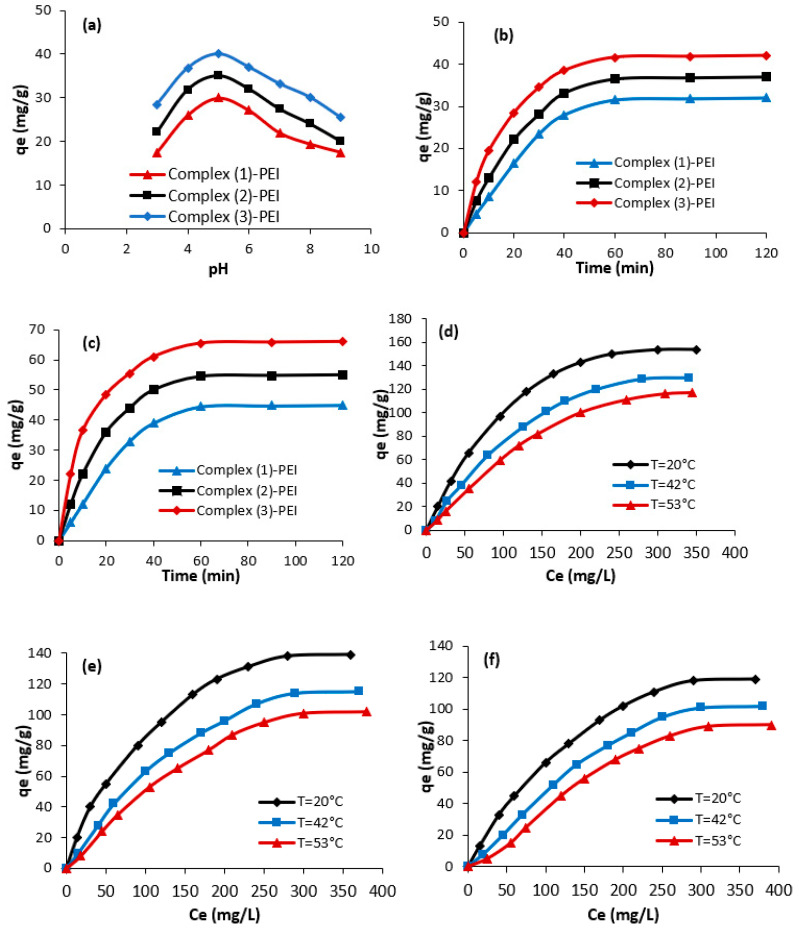
Influence of some experimental conditions on the adsorption of Naphthol blue black B using complex **(1)**-PEI, complex **(2)**-PEI, and complex **(3)**-PEI: (**a**) pH (C0 = 50 mg/L, T = 19 °C, time = 100 min), (**b**,**c**) time (C0 = 50 mg/L, C0 = 100 mg/L, pH = 5, T = 19 °C), and (**d**–**f**) temperature (data is replicated 3 times).

The prepared complexes **(1**–**3)** and the modified complexes were studied for the adsorption of Naphthol blue black B from water. The effects of changing pH, dye concentration, duration, and temperature on adsorption capabilities were examined. First, it is noticed that the adsorption capacities of Naphthol blue black B using the prepared complexes **(1**–**3)** are poor. However, complex **(1)**-PEI, complex **(2)**-PEI, and complex **(3)**-PEI showed high adsorption capacities. The next sections cover the adsorption of Naphthol blue black B utilizing three adsorbents: complex **(1)**-PEI, complex **(2)**-PEI, and complex **(3)**-PEI. Figure 7a shows the change in the adsorbed amounts of Naphthol blue black B against pH using complex **(1)**-PEI, complex **(2)**-PEI, and complex **(3)**-PEI. As observed, the adsorption capacities of Naphthol blue black B depend significantly on the pH value and it increase from 3 to 5.

At pH = 5, the three studied adsorbents reached their highest adsorption capabilities. At this pH, the negative charges of Naphthol blue black B molecules form strong electrostatic contacts with the positive charges on the surfaces of complexes **(1)**-PEI, **(2)**-PEI, and **(3)**-PEI. In acidic conditions, high concentrations of hydronium ions facilitate anionic dye adsorption. However, high concentrations of hydroxide ions in alkaline environments limit the adsorption capacity of anionic color molecules.

Figure 7b–d describe the change in the adsorption amounts of Naphthol blue black B using complex **(1)**-PEI, complex **(2)**-PEI, and complex **(3)**-PEI. As shown, the adsorption of Naphthol blue black B increases promptly with the reaction time (from 0 to 30 min). In fact, during this stage of adsorption, the number of adsorption sites on the surface of the studied adsorbents results in high adsorption capacity. After 60 min of reaction, the adsorption level tends to stabilize due to saturation of the available adsorption sites on the surfaces of complexes **(1)**-PEI, **(2)**-PEI, and **(3)**-PEI.

Figure 7d–f show the effect of the change in Naphthol blue black B concentration on the adsorption capacities. At low Naphthol blue black B concentrations, dye adsorption capabilities are readily achieved. This is owing to the accessibility of multiple adsorption sites at this phase, as well as the quick dispersion of dye from the original dye solution to the surface of complexes **(1)**-PEI, **(2)**-PEI, and **(3)**-PEI. At optimum adsorption conditions (T = 20 °C, time = 60 min, pH = 5), the highest adsorption capacities achieve 154 mg/g, 139 mg/g, and 119 mg/g for complex **(3)**-PEI, complex **(2)**-PEI, and complex **(1)**-PEI, respectively. The obtained results confirm the use of the prepared compounds as efficient adsorbents of Naphthol blue black B from water. In some cases, the obtained adsorption capacities are higher compared to many adsorbents studied in the literature, and in other cases, they are comparable to some adsorbents (Table 2). In fact, the chemical modification of the metalloporphyrinic complexes with polyethyleneimine greatly improves the adsorption of Naphthol blue black B due to the existence of reactive amine groups. As also observed in Figure 7d–f, the adsorption capacities vary as a function of temperature. The adsorption abilities were reduced with rising temperatures, indicating an exothermic phenomenon. The decrease in adsorption capabilities with temperature can be attributed to the partial breakdown of the association between Naphthol blue black B molecules and the modified metalloporphyrinic complex.

#### 3.2.2. Kinetic Investigation

The adsorption of Naphthol blue black B was evaluated using first order, second order, intra-particular dispersion, and Elovich kinetic models [48] for complexes **(1)**-PEI, **(2)**-PEI, and (3)-PEI. The computed variables are listed in Table 3. The high regression coefficients (R^2^ > 0.98) and correlation of measured adsorbed amounts of Naphthol blue black B with theoretically calculated amounts in the pseudo second order model imply a chemical adsorption mechanism [49,50]. The deviation from the origin in the intra-particular diffusion plots indicates that the adsorption of Naphthol blue black B is driven by both the intra-particular diffusion trend and other kinetic mechanisms [51].

#### 3.2.3. Determination of Thermodynamic Parameters

The three isotherms of Langmuir, Freundlich, and Temkin were evaluated in attempts to analyze the adsorption process of Naphthol blue black B employing the three adsorbents complex **(1)**-PEI, complex **(2)**-PEI, and complex **(3)**-PEI. Table 3 provides the fitting parameters and regression coefficients (R^2^). The high regression coefficients (R^2^ ≥ 0.97) indicate that the adsorption mechanism is consistent with the Freundlich and Temkin equations. According to these predictions, active adsorption sites may be unevenly distributed on the surfaces of complex **(1)**-PEI, complex **(2)**-PEI, and complex **(3)**-PEI [52].

**Figure 8:**
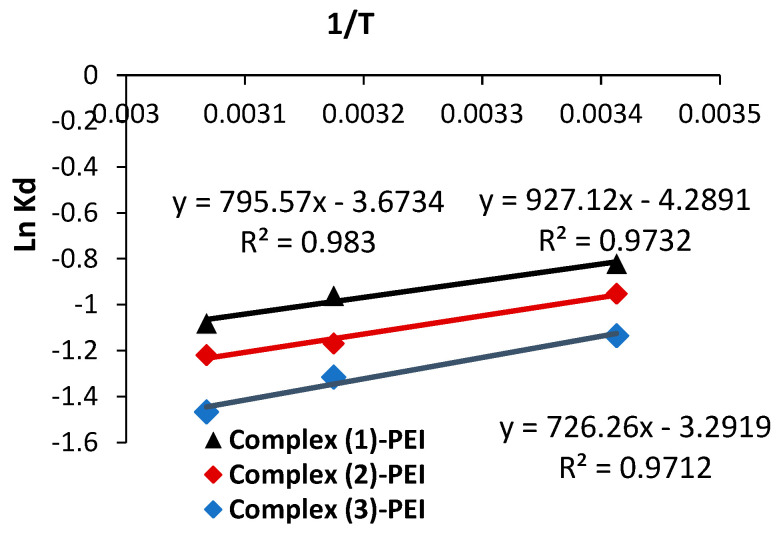
Plots of Ln K_d_ versus 1/T for Naphthol blue black B adsorption utilizing complexes **(1)**-PEI, **(2)**-PEI, and **(3)**-PEI (results replicated three times).

Figure 8 shows graphs of Ln (K_d_) vs. 1/T to derive the thermodynamic parameters (entropy and enthalpy). Table 3 shows the calculated values. The computed negative enthalpy values confirm that the adsorbent complexes **(1)**-PEI, **(2)**-PEI, and **(3)**-PEI underwent an exothermic reaction. As the temperature rises from 20 °C to 53 °C, the amount of adsorbed Naphthol blue black B molecules decreases, supporting this finding. The entropy values are negative, indicating that the disorder in the examined adsorption system has decreased, as well as the presence of many structural alterations [53]. The positive free energy values obtained during Naphthol blue black B adsorption show that the process is not spontaneous.

## 4. Conclusions

In this work, meso-tetrakis(2,4,6-trimethylphenyl) porphyrinato)zinc(II): ([Zn(TMP)] **(1)**, meso-tetrakis-(tetraphenyl)porphyrin iron(III))chloride): [Fe(TPP)Cl] **(2)**, and meso-tetrakis(phenyl)porphyrin manganese(III) chloride): [Mn(TPP)Cl] **(3)** were prepared. Then, the three prepared porphyrinic complexes **(1**–**3)** were chemically modified with branched polyethyleneimine (PEI). The synthesized compounds were examined using 1H NMR, FT-IR, UV-vis, XRD, XRF, TGA-DTA, SEM, and EDX. The presence of sharp peaks at 2θ between 10° and 80°, in XRD analysis, for all studied compounds suggested the crystalline nature of the porphyrinic complexes. The morphological properties of the porphyrinic complexes were significantly affected by the chemical modification with polyethyleneimine. EDX result confirmed the complexation of zinc, iron, and manganese metals with the porphyrinic core. The increase in carbon and nitrogen contents after the addition of polyethyleneimine to the complexes **1**–**3** was noticeable. After thermal decomposition, the total mass losses were equal to 92.97%, 66.77%, and 26.78% for complexes **1, 2,** and **3**, respectively. However, for the complex **(1)**-PEI, complex **(2)**-PEI, and complex **(3)**-PEI, the total mass losses were 83.12%, 81.88%, and 35.78%, respectively. The synthesized compounds were then utilized to adsorb Naphthol Blue Black B from water. At optimum adsorption conditions (T = 20 °C, time = 60 min, pH = 5), the highest adsorption capacities were 154 mg/g, 139 mg/g, and 119 mg/g for complex **(3)**-PEI, complex **(2)**-PEI, and complex **(1)**-PEI, respectively. The adsorption mechanism followed the pseudo second order, Freundlich, and Temkin models. The process was exothermic and non-spontaneous. Future studies will be extended for the design of other composites based porphyrinic complexes for the adsorption of other pollutants from contaminated water.

## Figures and Tables

**Table 1:** Relative abundance of elements present in complexes **1–3** and modified complexes.

m/m (%)						
Elements	Complex (1)	Complex (1)-PEI	Complex (2)	Complex (2)-PEI	Complex (3)	Complex (3)-PEI
Na	51.52	48.69	-	-	-	-
K	05.14	08.96	-	-	-	-
Zn	20.63	19.75	-	-	-	-
Al	19.20	18.59	-	-	-	-
Ca	01.70	01.59	-	-	-	-
Hf	01.14	01.05	-	-	-	-
Ti	00.67	01.37	-	-	-	-
Fe	-	-	52.60	51.93	-	-
Cl	-	-	40.17	39.58	-	-
Sx	-	-	06.02	07.01	-	-
V	-	-	00.69	00.71	-	-
Ni	-	-	00.44	00.70	-	-
Nb	-	-	00.02	00.05	-	-
Mo	-	-	00.10	00.02	-	-
Mn	-	-	-	-	70.59	68.70
Cl	-	-	-	-	28.60	27.25
Co	-	-	-	-	0.706	01.14
Nb	-	-	-	-	0.044	01.05
Mo	-	-	-	-	0.035	01.04
In	-	-	-	-	0.016	00.07

**Table 2:** Comparison of the adsorption capacities of Naphthol blue black B using some studied adsorbents.

	Q Max (mg/g)	Reference
**Complex (3)-PEI**	154	This study
**Complex (2)-PEI**	139	This study
**Complex (1)-PEI**	119	This study
**H_3_PO_4_ activated carbon produced from oak cupules**	208	[45]
**Immobilized Chitosan from Shrimp Shells onto Glass Plate**	45.5	[46]
**Commercial powdered activated carbon**	70.6	[47]

**Table 3:** Kinetics, isotherms, and thermodynamic parameters computed during the adsorption of Naphthol blue black B employing complexes **(1)**-PEI, **(2)**-PEI, and **(3)**-PEI.

	Constants	Naphthol Blue Black B (mg/L)		Isotherms	Parameters	Temperature (°C)		
**Complex (1)-PEI**								
**First order**		50	100			20	42	53
	K_1_ (min^−1^)	0.029	0.0306		q_m_ (mg·g^−1^)	222.22	250	243.90
	q (mg·g^−1^)	45.74	66.99	Langmuir	K_L_ (L·g^−1^)	0.0078	0.0046	0.39
	R^2^	0.98	0.98		R^2^	0.98	0.85	0.90
**Second order**	K_2_	0.0017	0.0005	Thermodynamic parameters	ΔH° (KJ mol^−1^)	−6.038		
	q	47.39	60.97		ΔS° (J mol^−1^)	−27.35		
	R^2^	0.99	0.99		ΔG° (KJ mol^−1^)	8.014	8.62	8.89
				Freundlich	K_F_ (L·g^−1^)	28.84	2.40	1.38
**Elovich**	*α* (mg·g^−1^·min^−1^)	32.06	45.37		n	1.54	1.24	1.21
	*β* (mg·g^−1^·min^−1^)	0.102	0.072		R^2^	0.97	0.97	0.98
	R^2^	0.95	0.95	Temkin	b_T_ (J·mol^−1^)	51.17	64.36	71.76
**Intra-particular-Diffusion**	K (mg·g^1^·min^1/2^)	3.36	4.73		A (L·g^−1^)	11.50	13.34	15.99
	R^2^	0.88	0.88		R^2^	0.98	0.97	0.97
**Complex (2)-PEI**								
**First order**	K_1_ (min^−1^)	0.029	0.031		q_m_ (mg·g^−1^)	188.67	204.08	227.27
	q (mg·g^−1^)	47.86	68.23	Langmuir	K_L_ (L·g^−1^)	0.0088	0.0042	0.392
	R^2^	0.98	0.98		R^2^	0.99	0.95	0.82
**Second order**	K_2_	0.0011	0.0009	Thermodynamic parameters	ΔH° (KJ mol^−1^)	−6.61		
	q	44.84	64.51		ΔS° (J mol^−1^)	−30.51		
	R^2^	0.99	0.99		ΔG° (KJ mol^−1^)	8.94	8.94	8.94
				Freundlich	K_F_ (L·g^−1^)	40.74	2.75	1.24
**Elovich**	*α* (mg·g^−1^·min^−1^)	23.49	27.43		n	1.67	1.30	1.19
	*β* (mg·g^−1^·min^−1^)	0.096	0.068		R^2^	0.97	0.97	0.97
	R^2^	0.95	0.94	Temkin	b_T_ (J·mol^−1^)	60.86	55.02	78.04
**Intra-particular-Diffusion**	K (mg·g^1^·min^1/2^)	3.75	5.47		A (L·g^−1^)	10.086	8.24	19.21
	R^2^	0.89	0.87		R^2^	0.97	0.97	0.97
**Complex (3)-PEI**								
**First order**	K_1_ (min^−1^)	0.0294	0.032		q_m_ (mg·g^−1^)	212.76	285.71	217.39
	q (mg·g^−1^)	45.92	68.55	Langmuir	K_L_ (L·g^−1^)	0.0038	0.0018	0.37
	R^2^	0.98	0.98		R^2^	0.95	0.68	0.73
**Second order**	K_2_	0.0017	0.015	Thermodynamic parameters	ΔH° (KJ mol^−1^)	−7.71		
	q	47.39	72.46		ΔS° (J mol^−1^)	−35.66		
	R^2^	0.99	0.99		ΔG° (KJ mol^−1^)	10.45	11.24	11.59
				Freundlich	K_F_ (L·g^−1^)	6.92	2.51	39.81
**Elovich**	*α* (mg·g^−1^·min^−1^)	23.43	27.20		n	1.43	1.12	1.01
	*β* (mg·g^−1^·min^−1^)	0.098	0.070		R^2^	0.97	0.97	0.97
	R^2^	0.94	0.94	Temkin	b_T_ (J·mol^−1^)	65.73	71.82	75.66
**Intra-particular-Diffusion**	K (mg·g^1^·min^1/2^)	4.007	6.02		A (L·g^−1^)	14.10	21.98	29.57
	R^2^	0.87	0.84		R^2^	0.97	0.97	0.97

## Data Availability

The original contributions presented in this study are included in the article. Further inquiries can be directed to the corresponding authors.

## References

[1] O’Neill J.S., Kearney L., Brandon M.P., Pryce M.T. (2022). Design components of porphyrin-based photocatalytic hydrogen evolution systems: A review. Coord. Chem. Rev..

[2] Das R., Verma P.K., Nagaraja C.M. (2024). Design of porphyrin-based frameworks for artificial photosynthesis and environmental remediation: Recent progress and future prospects. Coord. Chem. Rev..

[3] Vaz B., Pérez-Lorenzo M. (2023). Unraveling structure–performance relationships in porphyrin-sensitized TiO_2_ photocatalysts. Nanomaterials.

[4] Imran M., Ramzan M., Qureshi A.K., Khan M.A., Tariq M. (2018). Emerging applications of porphyrins and metalloporphyrins in biomedicine and diagnostic magnetic resonance imaging. Biosensors.

[5] Ptaszyńska A.A., Trytek M., Borsuk G., Buczek K., Rybicka-Jasińska K., Gryko D. (2018). Porphyrins inactivate *Nosema* spp.. microsporidia. Sci. Rep..

[6] Varchi G., Foglietta F., Canaparo R., Ballestri M., Arena F., Sotgiu G., Fanti S. (2015). Engineered porphyrin loaded core-shell nanoparticles for selective sonodynamic anticancer treatment. Nanomedicine.

[7] Tsolekile N., Nelana S., Oluwafemi O.S. (2019). Porphyrin as diagnostic and therapeutic agent. Molecules.

[8] Hammerer F., Garcia G., Chen S., Poyer F., Achelle S., Fiorini-Debuisschert C., Maillard P. (2014). Synthesis and characterization of glycoconjugated porphyrin triphenylamine hybrids for targeted two-photon photodynamic therapy. J. Org. Chem..

[9] Dong X., Chen H., Qin J., Wei C., Liang J., Liu T., Lv F. (2017). Thermosensitive porphyrin-incorporated hydrogel with four-arm PEG-PCL copolymer(II): Doxorubicin loaded hydrogel as a dual fluorescent drug delivery system for simultaneous imaging tracking in vivo. Drug Deliv..

[10] Zhang W., Taheri-Ledari R., Ganjali F., Mirmohammadi S.S., Qazi F.S., Saeidirad M., KashtiArayb A., Zarei-Shokatb S., Tianc Y., Maleki A. (2023). Effects of morphology and size of nanoscale drug carriers on cellular uptake and internalization process: A review. RSC Adv..

[11] Jenkins S.V., Srivatsan A., Reynolds K.Y., Gao F., Zhang Y., Heyes C.D., Chen J. (2016). Understanding the interactions between porphyrin-containing photosensitizers and polymer-coated nanoparticles in model biological environments. J. Colloid Interface Sci..

[12] Huang H., Song W., Rieffel J., Lovell J.F. (2015). Emerging applications of porphyrins in photomedicine. Front. Phys..

[13] Dini D., Calvete M.J., Hanack M. (2016). Nonlinear optical materials for the smart filtering of optical radiation. Chem. Rev..

[14] Charisiadis A., Nikolaou V., Nikoloudakis E., Ladomenou K., Charalambidis G., Coutsolelos A.G. (2025). Metalloporphyrins in bio-inspired photocatalytic conversions. Chem. Commun..

[15] Ding Y., Zhu W.H., Xie Y. (2017). Development of ion chemosensors based on porphyrin analogues. Chem. Rev..

[16] Leng F., Liu H., Ding M., Lin Q.P., Jiang H.L. (2018). Boosting Photocatalytic Hydrogen Production of Porphyrinic MOFs: The Metal Location in Metalloporphyrin Matters. ACS Catal..

[17] Zucca P., Neves C., Simões M.M., Neves M.D.G.P., Cocco G., Sanjust E. (2016). Immobilized lignin peroxidase-like metalloporphyrins as reusable catalysts in oxidative bleaching of industrial dyes. Molecules.

[18] Rabiee N., Yaraki M.T., Garakani S.M., Ahmadi S., Lajevardi A., Bagherzadeh M., Rabiee M., Tayebi L., Tahriri M., Hamblin M.R. (2020). Recent advances in porphyrin-based nanocomposites for effective targeted imaging and therapy. Biomaterials.

[19] Soury R., Alenezi K.M., Jabli M., Haque A., Al Otaibi A., El Moll H., Philouze C. (2021). Synthesis and characterization of axially modified Zn (II) porphyrin complexes for methylene blue dye oxidative degradation. J. Mol. Struct..

[20] Soury R., Jabli M., Saleh T.A., Kechich A., Loiseau F., Saint-Aman E., Nasri H. (2019). Degradation of Calmagite by dichloride (5, 10, 15, 20tetraphenylporphyrinato) antimony hexachloridoantimonate: [Sb (TPP) Cl_2_] SbCl_6_. Inorg. Chem. Commun..

[21] Nikoloudakis E., López-Duarte I., Charalambidis G., Ladomenou K., Ince M., Coutsolelos A.G. (2022). Porphyrins and phthalocyanines as biomimetic tools for photocatalytic H_2_ production and CO_2_ reduction. Chem. Soc. Rev..

[22] Grössl D.M., Hafner A.V., Fischer R.C., Saf R., Torvisco A., Uhlig F. (2023). Bis (chlorido) tin (IV) meso-substituted Porphyrins-Characterization and Solubility. Eur. J. Inorg. Chem..

[23] Lismont M., Dreesen L., Wuttke S. (2017). Metal-organic framework nanoparticles in photodynamic therapy: Current status and perspectives. Adv. Funct. Mater..

[24] Liang Z., Wang H.Y., Zheng H., Zhang W., Cao R. (2021). Porphyrin-based frameworks for oxygen electrocatalysis and catalytic reduction of carbon dioxide. Chem. Soc. Rev..

[25] Soury R., Chaabene M., Haque A., Jabli M., Alenezi K.M., Latif S., Abdulaziz F., Bchetnia A., Philouze C. (2022). Two novel pyrazine Zn (II)-porphyrins complexes: Synthesis, photophysical properties, structure study, DFT-Calculation and assessment of an azo dye removal from aqueous solution. J. Solid State Chem..

[26] Soury R., Jabli M., Saleh T.A., Abdul-Hassan W.S., Saint-Aman E., Loiseau F., Philouze C., Nasri H. (2018). (ethyl-4 (4-butyryl) oxyphenyl) porphyrinato zinc complexes with 4, 4′-bpyridin: Synthesis, characterization, and its catalytic degradation of Calmagite. RSC Adv..

[27] Soury R., Chaabene M., Jabli M., Saleh T.A., Chaabane R.B., Saint-Aman E., Loiseau F., Philouze C., Allouche A.-R., Nasri H. (2019). Meso-tetrakis (3, 4, 5-trimethoxyphenyl) porphyrin derivatives: Synthesis, spectroscopic characterizations and adsorption of NO_2_. Chem. Eng. J..

[28] Soury R., Alhar M.S., Jabli M. (2023). Synthesis, Characterization, and Application of Dichloride (5,10,15,20-Tetraphenylporphyrinato) Antimony Functionalized Pectin Biopolymer to Methylene Blue Adsorption. Polymers.

[29] Soury R., Jabli M., Latif S., Alenezi K.M., El Oudi M., Abdulaziz F., Teka S., El Moll H., Haque A. (2022). Synthesis and characterization of a new meso-tetrakis (2,4,6-trimethylphenyl) porphyrinto) zinc(II) supported sodium alginate gel beads for improved adsorption of methylene blue dye. Int. J. Biol. Macromol..

[30] Teng L., Yue C., Zhang G. (2023). Epoxied SiO_2_ nanoparticles and polyethyleneimine (PEI) coated polyvinylidene fluoride (PVDF) membrane for improved oil water separation, anti-fouling, dye and heavy metal ions removal capabilities. J. Colloid Interface Sci..

[31] Lang J.Q., Li C., Chen L., Mai T., Guo Z.H., Ma M.G. (2025). A polyethyleneimine-functionalized cellulose nanofiber/MXene composite aerogel: Towards highly efficient adsorption of crude oil, organic solvents, and dyes. J. Water Process Eng..

[32] Yan B., Dai Y., Li Y., Xin L., Li M., Long H., Gao X. (2025). Preparation of polyethyleneimine modified cellulose/nano-CdS composite aerogel and its photocatalytic properties for organic dyes under visible light. Int. J. Biol. Macromol..

[33] Soury R., Jabli M., Al Otaibi A. (2025). Rapid removal of anionic dyes from water, using poly (diallyldimethylammonium chloride) and branched polyethyleneimine functionalized cellulose extracted from *Echinops bannaticus* leaves. Results Chem..

[34] Soury R., Jabli M., Alenezi K.M., Haque A., Moll H.E., Rein R., Solladié N., Azzam E.M.S., Nasri H. (2021). A novel meso-tetrakis(2,4,6-trimethylphenyl) porphyrinato ([Zn(TMP)(4,4’-bpy)]) complex: Synthesis, characterization, and its performance for oxidative degradation of calmagite. Inorg. Chem. Commun..

[35] Alzabny M.H., Soury R., Alenezi K.M. (2021). Mn(III) and Fe(III) Porphyrin Complexes as Electrocatalysts for Hydrogen Evolution Reaction: A comparative study. Int. J. Electrochem. Sci..

[36] Soury R., Elamri A., El Oudi M., Alenezi K.M., Jabli M., Al Otaibi A., Alanazi A.A., Albadri A.E.A.E. (2024). Design of a New Catalyst, Manganese(III) Complex, for the Oxidative Degradation of Azo Dye Molecules in Water Using Hydrogen Peroxide. Molecules.

[37] Kurochkin I.Y., Olshevskaya V.A., Zaitsev A., Girichevac N., Girichev G. (2021). Vibrational Spectra of 5,10,15,20-Tetraphenylporphyrin (H2TPP) and Platinum(II) 5,10,15,20 Tetra(phenyl/pentafluorophenyl)porphyrins (PtTPP and PtTF5PP). Macroheterocycles.

[38] Soury R., Jabli M., Oudi M.E., Alenezi K.M., Otaibi A.A., Abdulaziz F., Al Ghamdi H.A., Bchetnia A. (2025). New complexes of [5,10,15,20-(tetraphenylporphyrin)] and dichloride (5,10,15,20-tetraphenylporphyrinato) antimony(V) hexachloridoantimonate(V) functionalized with polyethyleneimine: Synthesis, characterization, and application in Eriochrome Black T adsorption from water. Polyhedron.

[39] Jabli M., Sebeia N., El-Ghoul Y., Soury R., Al-Ghamdi Y.O., Saleh T.A. (2023). Chemical modification of microcrystalline cellulose with polyethyleneimine and hydrazine: Characterization and evaluation of its adsorption power toward anionic dyes. Int. J. Biol. Macromol..

[40] Gao W.Y., Chrzanowski M., Ma S. (2014). Metal–metalloporphyrin frameworks: A resurging class of functional materials. Chem. Soc. Rev..

[41] Gamelas S.R., Tomé J.P., Tomé A.C., Lourenço L.M. (2024). Porphyrin-containing materials for photodegradation of organic pollutants in wastewaters: A review. Catal. Sci. Technol..

[42] Bao Y. (2024). Polymerization-Mediated Through-Space Charge Transfer: An Emerging Strategy for Light-Emitting Materials. Langmuir.

[43] Chen M., Li H., Liu C., Liu J., Feng Y., Wee A.G., Zhang B. (2021). Porphyrin-and porphyrinoid-based covalent organic frameworks (COFs): From design, synthesis to applications. Coord. Chem. Rev..

[44] Trache D., Donnot A., Khimeche K., Benelmir R., Brosse N. (2014). Physicochemical properties and thermal stability of microcrystalline cellulose isolated from Alfa fibres. Carbohydr. Polym..

[45] Manal Alkhabbas Alaa M. (2023). Al-Ma’abreh, Gada Edris, Tasneem Saleh, Heba Alhmood. Adsorption of Anionic and Cationic Dyes on Activated Carbon Prepared from Oak Cupules: Kinetics and Thermodynamics Studies. Int. J. Environ. Res. Public Health.

[46] Fathurrahmi F., Robbani F. (2022). Adsorption of Naphtol Blue Black (NBB) Dye over Immobilized Chitosan from Shrimp Shells onto Glass Plate. J. Pharm. Sci..

[47] Benammar H.S., Guergazi S., Youcef S., Youcef L. (2021). Removal of Congo red and Naphthol blue black dyes from aqueous solution by adsorption on activated carbon. Characterization, kinetic and equilibrium in nonlinear models studies. Desalin. Water Treat..

[48] Costescu A., Pasuk I., Ungureanu F., Dinischiotu A., Costache M., Huneau F., Galaup S., Le Coustumer P., Predoi D. (2010). Physico-chemical properties of nano-sized hexagonal hydroxyapatite powder synthesized by sol-gel. Dig. J. Nanomater. Biostruct..

[49] Bulina N.V., Makarova S.V., Baev S.G., Matvienko A.A., Gerasimov K.B., Logutenko O.A., Bystrov V.S. (2021). A Study of Thermal Stability of Hydroxyapatite. Minerals.

[50] Einhorn-Stoll U., Kunzek H., Dongowski G. (2007). Thermal analysis of chemically and mechanically modified pectins. Food Hydrocoll..

[51] Wei W., Yang L., Zhong W.H., Li S.Y., Cui J., Wei Z.G. (2015). Fast removal of methylene blue from aqueous solution by adsorption onto poorly crystalline hydroxyapatite nanoparticles. Dig. J. Nanomater. Biostruct..

[52] Zhang J., Ping Q., Niu M., Shi H., Li N. (2013). Kinetics and equilibrium studies from the methylene blue adsorption on diatomite treated with sodium hydroxide. Appl. Clay Sci..

[53] Nasuha N., Hameed B.H., Din A.T. (2010). Rejected tea as a potential low-cost adsorbent for the removal of methylene blue. J. Hazard. Mater..

